# TRPA1 mediates amplified sympathetic responsiveness to activation of metabolically sensitive muscle afferents in rats with femoral artery occlusion

**DOI:** 10.3389/fphys.2015.00249

**Published:** 2015-09-15

**Authors:** Jihong Xing, Jian Lu, Jianhua Li

**Affiliations:** ^1^Department of Emergency Medicine, The First Hospital of Jilin UniversityChangchun, Jilin, China; ^2^Pennsylvania State Heart and Vascular Institute, The Pennsylvania State University College of MedicineHershey, PA, USA

**Keywords:** muscle afferent nerve, TRPA1, peripheral arterial disease, hindlimb ischemia

## Abstract

Autonomic responses to activation of mechanically and metabolically sensitive muscle afferent nerves during static contraction are augmented in rats with femoral artery occlusion. Moreover, metabolically sensitive transient receptor potential cation channel subfamily A, member 1 (TRPA1) has been reported to contribute to sympathetic nerve activity (SNA) and arterial blood pressure (BP) responses evoked by static muscle contraction. Thus, in the present study, we examined the mechanisms by which afferent nerves' TRPA1 plays a role in regulating amplified sympathetic responsiveness due to a restriction of blood flow directed to the hindlimb muscles. Our data show that 24–72 h of femoral artery occlusion (1) upregulates the protein levels of TRPA1 in dorsal root ganglion (DRG) tissues; (2) selectively increases expression of TRPA1 in DRG neurons supplying metabolically sensitive afferent nerves of C-fiber (group IV); and (3) enhances renal SNA and BP responses to AITC (a TRPA1 agonist) injected into the hindlimb muscles. In addition, our data demonstrate that blocking TRPA1 attenuates SNA and BP responses during muscle contraction to a greater degree in ligated rats than those responses in control rats. In contrast, blocking TRPA1 fails to attenuate SNA and BP responses during passive tendon stretch in both groups. Overall, results of this study indicate that alternations in muscle afferent nerves' TRPA1 likely contribute to enhanced sympathetically mediated autonomic responses via the metabolic component of the muscle reflex under circumstances of chronic muscle ischemia.

## Introduction

Peripheral arterial disease (PAD) is atherosclerotic disease with a decrease in blood flow to the arteries of the lower extremities. In this disease, the most common symptom is intermittent claudication, which is worsened by exercise activity due to muscle ischemia but subsides at when the metabolic demand of the active muscles is decreased (Rejeski et al., 2008).

Exercise increases sympathetic nerve activity (SNA) (Mark et al., 1985; Victor et al., 1988; Sinoway et al., 1989), an effect which in turn increases arterial blood pressure (BP), heart rate (HR), myocardial contractility and peripheral vascular resistance. Two mechanisms, namely central command and the exercise pressor reflex, evoke this exercise-induced increase in SNA. Central command postulates a parallel and simultaneous increase in sympathetic and alpha motoneuron discharge (Goodwin et al., 1972; Waldrop et al., 1996). The exercise pressor reflex postulates that thin fiber muscle afferent nerves (group III & IV) innervating skeletal muscles are activated by contraction-induced mechanical and metabolic stimuli to elicit a reflex increase in SNA (Mccloskey and Mitchell, 1972; Mitchell et al., 1983; Kaufman and Forster, 1996). It was observed that both systolic and diastolic BP rise significantly in the patients with PAD than in the normal subjects during walking (Baccelli et al., 1997). Furthermore, the exercise pressor reflex plays a crucial role in evoking the exaggerated BP response to walking in PAD patients (Baccelli et al., 1999).

Ligation of the femoral artery in rats serves as a useful model to study human PAD (Waters et al., 2004). Using this model, prior studies have shown that the SNA and BP responses to muscle contraction as well as to stimulation of several muscle metabolic receptors are enhanced in ligated rats compared with non-ligated rats (Li and Xing, 2012). Nonetheless, the precious mechanisms responsible for amplified responsiveness of SNA and BP during stimulation of muscle afferents in ligated animals still need to be determined.

Transient receptor potential channel A1 (TRPA1) is a member of branch A of the transient receptor potential (TRP) family of nonselective cation channels. This channel is expressed in the sensory (nerves) neurons and is involved in acute and inflammatory pain (Story et al., 2003; Bandell et al., 2004; Obata et al., 2005; Bautista et al., 2006; Katsura et al., 2006; Kwan et al., 2006; Macpherson et al., 2007). TRPA1 acts as a sensory receptor in response to pungent and reactive chemicals such as allylisothiocyanate (AITC, used as a TRPA1 agonist), allicin, cinnamaldehyde, formaldehyde, N-methylmaleimide, and α, β-unsaturated aldehydes (Story et al., 2003; Bandell et al., 2004; Jordt et al., 2004; Bautista et al., 2005). TRPA1 also serves as a sensor of cold temperature and mechanical deformation (Story et al., 2003; Nagata et al., 2005; Obata et al., 2005; Kwan et al., 2006; Kindt et al., 2007; Sawada et al., 2007; Trevisani et al., 2007). Besides pungent chemicals, endogenously generated molecules i.e., bradykinin, reactive oxygen species, and 4-hydroxynonenal that are produced during inflammation and oxidative stress, respectively, can activate TRPA1 (Bandell et al., 2004; Trevisani et al., 2007; Bessac et al., 2008).

A recent study has demonstrated that intra-arterial injection of AITC into the hindlimb muscle circulation of healthy rats led to increases in SNA and BP via a reflex mechanism (Koba et al., 2011). Also, this study has suggested that TRPA1 plays a role in activating the exercise pressor reflex and acid phosphate, bradykinin and arachidonic acid, which are accumulated in exercising muscle, are likely endogenous stimulants of TRPA1.

Thus, the purpose of this study was to determine the mechanisms by which afferent nerves' TRPA1 contributes to the regulation of the amplified sympathetic responsiveness in a rat model of femoral artery occlusion. Overall, our hypothesis was that the higher protein levels of TRPA1 in dorsal root ganglion (DRG) neurons supplying metabolically sensitive afferent nerves of C-fiber (group IV) are induced with a restriction of blood flow directed to the hindlimb muscles, effects which in turn exaggerate the metabolic component of the exercise pressor reflex as a consequence.

## Materials and methods

The Institutional Animal Care and Use Committee of Pennsylvania State College of Medicine has approved all animal experimental procedures, which were complied with the National Institutes of Health (NIH) guidelines.

### Ligation of femoral artery

Under inhalation of an isoflurane-oxygen mixture (2–5% isoflurane in 100% oxygen), the surgical procedures were performed in 86 male Sprague–Dawley rats (5–7 week old) as previously described (Xing et al., 2008; Liu et al., 2010; Lu et al., 2013). For the western blotting and (*n* = 18) immunofluorescence experiments (*n* = 4), the rat's femoral artery on one limb was surgically exposed, dissected, and ligated ~3 mm distal to the inguinal ligament; this served as “ligated limb.” The same procedures were performed on the other limb except that a suture was placed below the femoral artery but was not tied; this served as “control limb.” For the experiment of SNA and BP recording, the rats were divided between those that had the right femoral artery ligation [“ligated rats” (*n* = 34)] and those that had sham surgeries on the right hindlimb [“control rat” (*n* = 30)]. Then, 6 to 72 h were allowed for recovery before the experiments began.

### Western blot analysis

Eighteen rats were used to examine expression of TRPA1 protein in lumbar (L4–L6) DRGs of control and limbs with 6, 24, and 72 h of femoral artery occlusion. Western blot methods were performed as previously described (Liu et al., 2010; Lu et al., 2013). In brief, DRGs of the rats were removed. All DRGs tissues from individual rats were sampled for western blot analysis. Total protein was then extracted by homogenizing DRG sample in ice-cold radioimmunoprecipitation assay buffer containing 25 mM Tris·HCl (pH 7.6), 150 mM NaCl, 1% Nonidet P-40, 1% sodium deoxycholate, and 0.1% sodium dodecyl sulfate (SDS) with protease inhibitor cocktail kit (Sigma-Aldrich, St. Louis, MO). The lysates were centrifuged at 15,000 g for 15 min at 4°C; the supernatants were collected for measurements of protein concentrations using a bicinchoninic acid assay reagent kit (Pierce Biotechnology, Rockford, IL) and then stored in −80°C for later use.

After being denatured by heating at 95°C for 5 min in an SDS sample buffer (Cell Signaling Technology, Danvers, MA), the supernatant samples containing 20 μg of protein was loaded onto 4–20% Mini-Protean TGX Precast gels (Bio-Rad Laboratories, Hercules, CA) and then electrically transferred to a polyvinylidene fluoride membrane (GE Water & Process Tech, Trevose, PA) (Liu et al., 2010; Lu et al., 2013). The membrane was blocked in 5% nonfat milk in 0.1% Tween-TBS buffer for 1 h and was then incubated overnight with primary antibody: rabbit anit-TRPA1 at 1:500 dilutions (Novus).

After being fully washed, the membrane was incubated with horseradish peroxidase-linked anti-rabbit secondary antibody at 1:1000 dilutions and visualized for immunoreactivity using an enhanced chemiluminescence system (Cell Signaling Technology). The membrane was stripped and incubated with mouse anti-β-actin (Sigma-Aldrich) to show equal loading of the protein in the western blot analysis. The bands recognized by the primary antibody were visualized by exposure of the membrane onto an X-ray film. Then, the film was scanned and the optical densities of TRPA1 and β-actin bands were determined using the NIH Scion Image Software (Liu et al., 2010; Lu et al., 2013).

### Fluorescence immunohistochemistry

As described in our previous work (Liu et al., 2010; Lu et al., 2012; Xing et al., 2012), the four rats were anesthetized by inhalation of an isoflurane-oxygen mixture and then transcardially perfused with 200 ml of ice-cold saline containing 1000 units heparin followed by 500 ml of 4% fresh prepared, ice-cold paraformaldehyde in phosphate-buffered saline (PBS, pH 7.4). L4–L6 DRGs of control limbs and limbs with 24 h of femoral occlusion were immediately dissected out and immersed in the same fixative at 4°C for 2 h. The tissues were then stored in PBS containing 30% sucrose overnight. Then, a cryostat was used to obtain 10 μm of DRG sections.

DRG sections were fixed in 4% of paraformaldehyde in PBS for 10 min at room temperature. After being washed with PBS, the tissue were permeabilized, blocked in 0.3% Triton X-100 in PBS supplemented with 5% goat serum for 1 h, and then incubated with rabbit anti-TRPA1 (1:200, Novus) antibody overnight at 4°C. After being washed in PBS, the sections were incubated with goat anti-rabbit fluorescein isothiocyanate (FITC) labeled secondary antibody (1: 200, Invitrogen) for 2 h at room temperature.

To examine localization of TRPA1 within DRG neurons supplying C-fiber and A-fiber, the sections were incubated with the second primary antibody (mouse anti-peripherin at 1:200, Sigma; and anti-NF200 at 1:200 Abcam) overnight (Liu et al., 2010; Lu et al., 2012; Xing et al., 2012). Peripherin and NF200 are used to label neurons with C-fiber and A-fiber, respectively. Briefly, after incubation, the sections were washed and incubated for 2 h at room temperature with secondary antibody (Alexa Fluor-594 conjugated goat anti-mouse IgG, dilution: 1:200). Then, the sections were washed in PBS, and coverslipped.

FITC- and Alexa Fluor-594-labeled DRG neurons were examined using a Nikon Eclipse 80i microscope with appropriate filters, and the images were stored digitally on a computer. As described previously (Liu et al., 2010), at least five sections containing L4–L6 DRGs per rat were randomly chosen for analysis of FITC and Alexa Fluor-594 staining intensity. Thus, the four rats used in this experiment provided a sufficient power to statistical analysis. A threshold value of staining intensity was set according to the mean staining intensity of background using the Nis-Elements software (Nikon, Co.). Cells with >1.75 times of background intensity were considered to be positive. The number of total TRPA1 immunostaining and peripherin/NF200 positive neurons was counted in each section (Liu et al., 2010). Percentages of double (FITC and peripherin /NF200)-labeled neurons were calculated: total number of double-labeled cells × 100/total number of peripherin/NF200 positive cells (Lu et al., 2012; Xing et al., 2012, 2013). Note that the majority of DRG neurons showed a clear nucleus and perimeter and they were counted. To minimize the possibility of counting a single DRG neuron more than once, DRG sections were collected on 5 glass slides in series, and tissues from one of slides were processed for immunocytochemical analysis.

### Examination of the exercise pressor reflex

#### Surgical procedures

The 30 control rats and 34 rats with 24 h of femoral occlusion were anesthetized with a mixture of 2–5% isoflurane and oxygen and ventilated as describe previously (Xing et al., 2008; Liu et al., 2010; Lu et al., 2013). The right jugular vein and common carotid artery were cannulated to deliver fluids and to connect a pressure transducer for measurement arterial BP, respectively. A catheter (PE10) was then inserted into the distal side of the right femoral artery for injection of drugs into the arterial blood supply of the hindlimb muscles. During the experiments, baseline BP and fluid balance were maintained with a continuous infusion of saline. Body temperature was continuously monitored and maintained at 37.5–38.5°C with a heating pad and external heating lamps.

A laminectomy was performed to expose the lower lumbar and upper scaral portions of the spinal cord after the rats were placed in a spinal unit (Kopf Instruments) as described in the previous work (Smith et al., 2001; Lu et al., 2012). The spinal roots were exposed and the right L4 and L5 ventral roots visually identified with assistance of an anatomical microscope (Cooper Surgical, Inc.). The peripheral ends of the transected L4 and L5 ventral roots were then placed on bipolar platinum stimulating electrodes. A pool was formed by using the skin and muscle on the back and the exposed spinal region was filled with warmed (37°C) mineral oil.

In a subset group of experiments, a bundle of the renal nerves on the left side was carefully dissected from other connective tissues. A piece of laboratory film was placed under the isolated nerves, and two tips of a bipolar electrode used to record neural activity were placed between the nerves and the film; these were embedded in a silicone gel. Once the gel hardened, the silicone rubber was fixed to the surrounding tissue with a glue containing α-cyanoacrylate. The skin on the back was used to form a pool that was filled with warm (37°C) mineral oil. The renal SNA (RSNA) signal was amplified with an amplifier (P511, Grass Instruments) with a band-pass filter of 300 Hz in low-cut frequency and of 3 kHz in high-cut frequency and recorded as previously described (Xing et al., 2008; Liu et al., 2010; Lu et al., 2013).

Decerebration was performed as previously described (Smith et al., 2001; Xing et al., 2008; Liu et al., 2010; Lu et al., 2012) to avoid the confounding effects of anesthesia on the reflex pressor response. A transverse section was made anterior to the superior colliculus and extending ventrally to the mammillary bodies. All brain tissues from rostral to the section were removed. Following this procedure, the anesthesia was withdrawn from the rats. The calcaneal bone of right hindlimb was cut and its tendon was attached to a force transducer (Grass FT10), and the knee joints were secured by clamping the patellar tendon to a spinal unit. A recovery period of 60 min was allowed before the experiment began.

#### Experimental protocols

**Figure 1:**
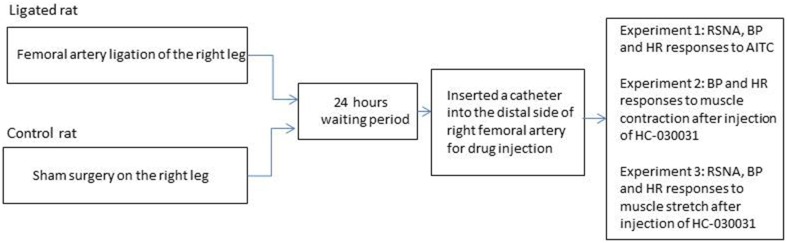
**This diagram illustrates experimental designs examining the exercise pressor reflex**.

A diagram of Figure 1 illustrates experimental designs examining the exercise pressor reflex. In the first group of experiments, in order to determine the sympathetic, BP and HR responses to activation of metabolically sensitive TRPA1 in control rats and ligated rats. Three dosages of AITC (TRPA1 agonist) (10, 20, 40 μg/kg body) were injected into arterial blood supply of the right hindlimb muscles of control rats (*n* = 12) and rats with 24 h of femoral artery occlusion (*n* = 14). The concentrations of AITC were selected on the basis of the results of a prior study (Koba et al., 2011). The injection volume was adjusted to 0.1–0.2 ml according to rat's body weight. The duration of the injection was 1 min. At least 20 min were allowed between injections. In some experiments, sciatic and femoral nerves were cut and then 40 μg/kg of AITC was injected in control rats and ligated rats (*n* = 5 in each group). This allowed us to rule out the systemic effects of AITC.

In the second group of experiments, static muscle contractions in the right hindlimb were performed by electrical stimulation of the L4 and L5 ventral roots (30 s, 3-times motor threshold with a period of 0.1 ms at 40 Hz) in control rats (*n* = 8) and rats whose femoral artery was ligated for 24 h (*n* = 8). The purpose of this experiment was to examine if femoral artery occlusion altered the effects of blocking TRPA1 receptor on BP and HR responses evoked by stimulation of both mechanically and chemically sensitive muscle afferent nerves. Note that RSNA was not examined in this experiment due to electrical interference during the ventral root stimulation. Each static muscle contraction was performed 15 min after arterial injection of DMSO (control and recovery) and 3 mg of HC-030031 (TRPA1 antagonist) (Koba et al., 2011). Then, 20 min was allowed after contraction and before next injection. The injected volume was 0.25 ml and the duration of injections was 1 min. Thus, there was a ~36-min resting period between bouts of muscle contraction. In addition, 3 mg of HC-030031 (0.25 ml over 1 min) was injected into jugular vein and then BP response to contraction was examined in six control rats. This allowed us to exclude the systemic effects evoked by arterial injection HC-030031.

In the third group of experiments, passive tendon stretch was performed in the right hindlimb of control rats (*n* = 10) and rats (*n* = 12) whose femoral artery was ligated for 24 h before the experiments. Passive tendon stretch (500 g of tension) was produced manually over ~5 s by using a rack and pinion attached to the Achilles' tendon of control and ligated rats. Each bout of muscle stretch was maintained for 30 s after 500 g of tension was achieved. Muscle stretch was used to activate the mechanoreceptor component of the exercise pressor reflex. The purpose of this study was to examine if vascular insufficiency observed in ligated rats altered SNA, BP, and HR responses evoked by stimulation of mechanically sensitive muscle afferent nerves alone. Similar to the protocol used in the muscle contraction experiments, arterial injection of DMSO before muscle stretch was performed as control and recovery and arterial injection of 3 mg of HC-030031 was employed to block TRPA1.

#### Recording of experimental data

All measured data of RSNA, BP, HR and muscle tension were continuously recorded and stored on a computer with PowerLab system (Ad instruments, Castle Hill, Australia). As described previously (Xing et al., 2008; Lu et al., 2012, 2013), mean arterial pressure (MAP) was obtained by integrating the arterial signal with a time constant of 4 s. HR was calculated on a basis of beat to beat from the arterial pressure pulse. The peak responses of MAP and HR were determined by the peak change from the control value. The muscle tension response during the ventral root stimulation was also recorded. RSNA signals were transformed into absolute values, integrated over 1 s interval, and subtracted by 1 s of integrated background noise. To quantify RSNA response to AITC injection and passive tendon stretch (by loading ~500 g of muscle tension), baseline values were obtained by taking the mean value for the 30 s immediately before each intervention and by ascribing the mean value of 100%, and relative change from baseline during the injection and stretch were then evaluated.

### Data statistical analysis

One-Way repeated measures analysis of variance (ANOVA) was used to analyze data of Western Blot and immunohistochemistry, and Two-Way ANOVA was used to analyze data of RSNA, MAP, HR, and muscle tension. As appropriate, Tukey's *post-hoc* tests were used. All values were presented as mean ± SEM. For all analyses, differences were considered significant at *P* < 0.05. All statistical analyses were performed using SPSS for Windows version 20.0.

## Results

### TRPA1 expression in DRG neurons

**Figure 2:**
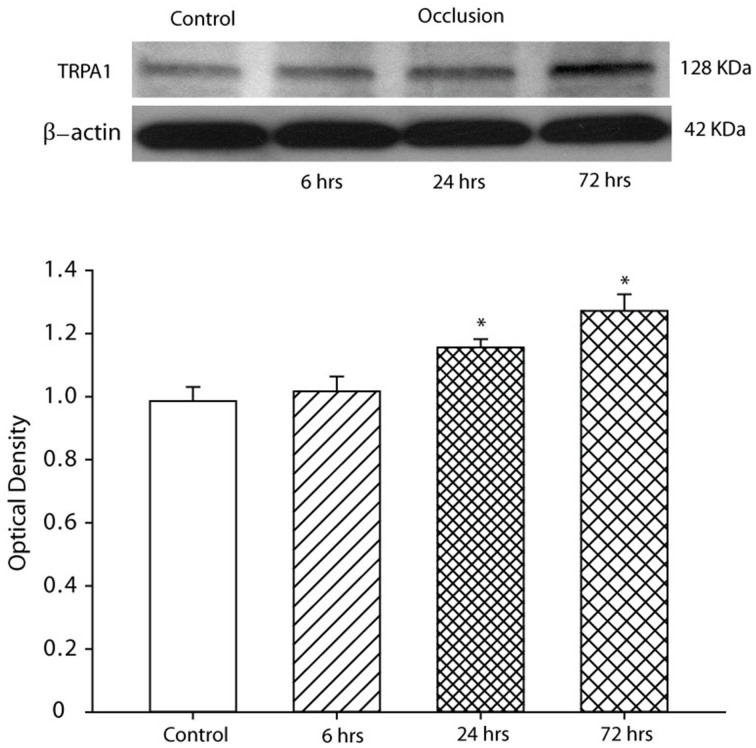
**Effects of femoral artery occlusion on TRPA1 expression at different time courses**. Western blot analysis was used to examine the protein levels of TRPA1 in DRG tissues. **Top**: representative bands of TRPA1 expression. Bands of β-actin are used as control for an equal protein loading. **Bottom**: average data. The optical density is expressed in arbitrary units normalized against a control sample. Data in histograms represent means ± SEM; *n* = 6 in each group. ^*^P < 0.05 vs. control.

The expression of TRPA1 proteins in L4–L6 DRG neurons of control and ligated limbs was examined using western blot analysis. Typical bands and average data of Figure 2 show that the protein levels of TRPA1 are significantly increased 24 and 72 h (*n* = 6 in each group) after femoral artery ligation. Nevertheless, 6 h of femoral artery ligation (*n* = 6) did not significantly increase TRPA1 expression as compared with control. In addition, there were insignificant differences in the protein levels of TRPA1 24 and 72 h after the ligation. Thus, 24 h of femoral artery ligation was used for other groups of experiments in this report.

### TRPA1 distributions within DRG neurons with different phenotypes

**Figure 3:**
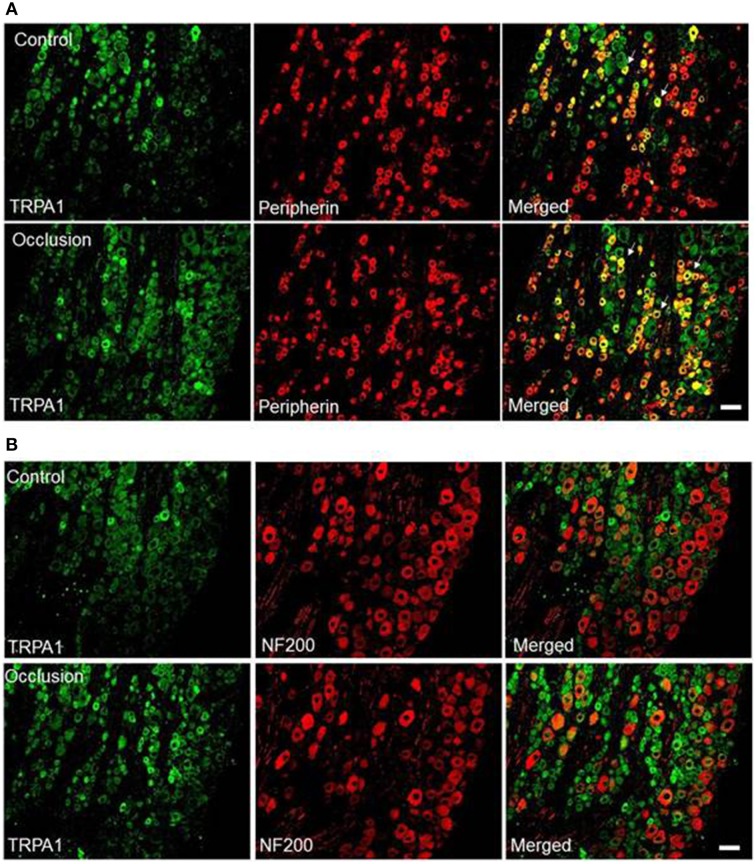
**Immunofluorescence was employed to examine double-labeling for TRPA1 and peripherin and NF200**. Peripherin was used to label DRG neurons that supply thin C-fibers afferent nerves. NF200 was used to identify A-fibers of DRG neurons. **(A)** Representative photomicrographs show TRPA1 and peripherin staining in DRG neurons of a control limb (top) and a ligated limb (bottom). Arrows indicate representative cells positive for both TRPA1 and peripherin after they were merged. The number of double labeled DRG neurons is greater in ligated limbs than in control limbs. Scale bar = 50 μm. **(B)** Photomicrographs are representative to illustrate staining of TRPA1 and NF200 in DRG neurons of a control limb (top) and a ligated limb (bottom). There were few DRG neurons containing both TRPA1 and NF200 staining in both groups. No differences in the number of double-stained TRPA1 and NF200 were observed in DRG neurons of the control and ligated limbs. Scale bar = 50 μm.

In this experiment, we further determined if TRPA1 exists within DRG neurons that supply C- and/or A-fiber afferent nerves. The dual immunofluorescence techniques were used to examine co-localization of fluorescent TRPA1 and peripherin/NF200 immunoreactivity in the DRG neurons of control limbs and ligated limbs. The appearance of TRPA1 and peripherin/NF200 within DRG neurons is characterized by fluorescent green and red color, respectively (Figures 3A,B). Note that there was no significant difference in number of peripherin- and NF200-positve DRG neurons between both experimental groups.

The photomicrographs in Figure 3A show that the most TRPA1 staining appears in C-fiber of DRG neurons in both control and ligated groups. The percentage of double-labeled neurons with TRPA1 and peripherin was significantly greater in the ligated limbs than that in control limbs. They were 39 ± 2% in control limbs (*n* = 4) and 52 ± 3% (*P* < 0.05 vs. control) in ligated limbs (*n* = 4). In contrast, the photomicrographs in Figure 3B show that few A-fiber of DRG neurons include TRPA1 staining in both control and ligated groups. The percentage of double-labeled neurons with TRPA1 and NF200 was similar in both groups. These double-labeled neuron were < 5% in controls (*n* = 4) and in the ligation group (*n* = 4) (*P* > 0.05 vs. control).

### Engagement of TRPA1 in the exercise pressor reflex

#### RSNA, BP, and HR responses to stimulation of TRPA1

**Figure 4:**
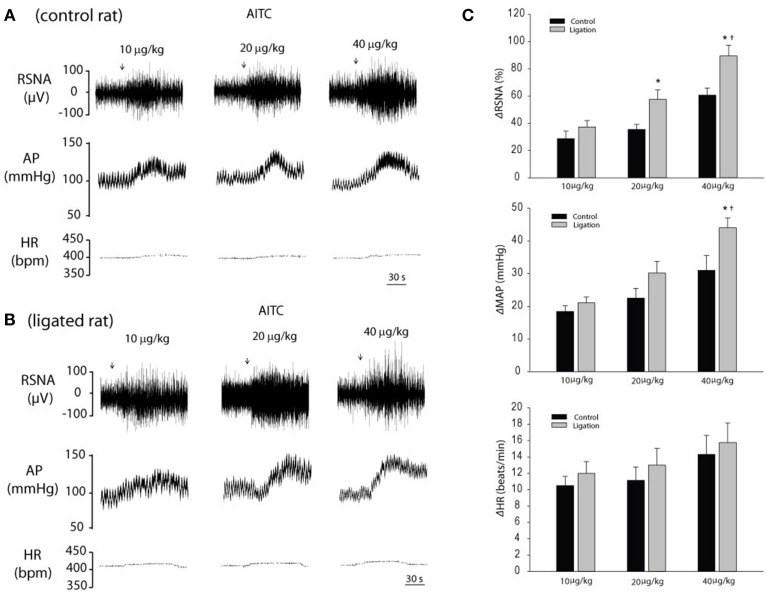
**Changes in RSNA, MAP and HR in response to stimulation of TRPA1 with arterial injection of AITC (TRPA1 agonist)**. **(A,B)** Typical traces obtained from a control rat and ligated rat. Arrows indicate a start of injections. **(C)** Average data. Three dosages of AITC (10, 20, 40 μg/kg body) were injected into the arterial blood supply of the hindlimb muscles of control rats (*n* = 12) and rats with 24 h of femoral artery occlusion (*n* = 14). No significant difference was observed in baseline MAP and HR. Values are means ± SEM. ^*^P < 0.05, compared with control. ^†^*P* < 0.05 indicates significant differences among different dosages.

Baseline values for MAP and HR before arterial injections of AITC are 93 ± 4 mmHg and 396 ± 16 beats/min in control rats (*n* = 12); and 91 ± 3 mmHg and 403 ± 19 beats/min in ligated rats (*n* = 14), respectively. There were no significant differences in basal MAP and HR before injections. Typical and average data of Figure 4 illustrates the effects of increasing concentrations (10, 20, and 40 μg/kg body wt) of AITC injected into the hindlimb muscles on RSNA, MAP, and HR in control rats and ligated rats. Arterial injection of AITC evoked dose-related increases in RSNA and MAP in both groups. Those responses induced by 40 μg/kg of AITC were significantly amplified in ligated rats compared with responses in control rats. The RSNA and MAP responses to arterial injection of AITC (40 μg/kg body wt) were 61 ± 5% and 31 ± 5 mmHg in 12 control rats; and 89 ± 8% and 44 ± 3 mmHg in 14 ligated rats (*P* < 0.05 ligation vs. control), respectively. Note that there was no significant difference in HR responses evoked by an arterial injection of AITC in both groups. In addition, MAP response to arterial injection of 40 μg of AITC was 4 ± 1 mmHg in control rats (*n* = 5) and 5 ± 1 mmHg in ligated rats (*n* = 5) after section of the sciatic and femoral nerves (*P* > 0.05 vs. their respective baseline).

#### BP and HR responses to muscle contraction after blocking of TRPA1

**Figure 5:**
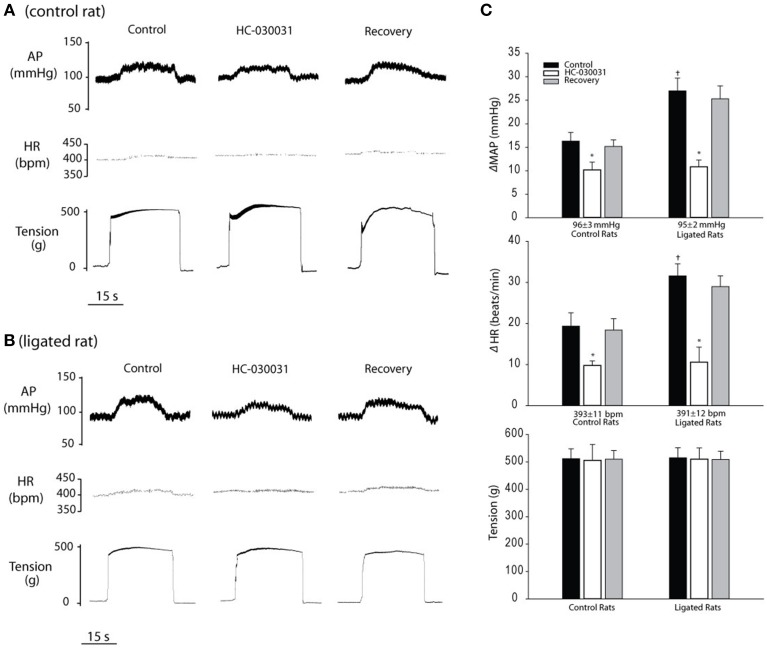
**Changes in MAP, HR and muscle tension in response to static contraction after an inhibition of TRPA1 by 3 mg of HC-030031 (TRPA1 antagonist)**. Muscle contraction was evoked by electrical stimulation of the L4 and L5 ventral roots. Blocking TRPA1 significantly attenuated the MAP and HR responses to muscle contraction in control rats and rats with 24 h of femoral artery ligation. **(A,B)** Typical traces obtained from a control rat and ligated rat. **(C)** Average data. The attenuating effects of HC-030031 were greater in ligated rats (MAP and HR: 59 and 67%. *P* < 0.05 vs. control rats, *n* = 8) than in control rats (MAP and HR: 38 and 49%, *n* = 8). No significant difference was observed in baseline MAP and HR, and muscle tension responses in two groups. Values are means ± SEM. ^*^P < 0.05 vs. vehicle control. ^†^*P* < 0.05 indicates ligated rats vs. control rats.

Baseline MAPs and HRs were 96 ± 3 mm Hg and 393 ± 11 bpm in control rats (*n* = 8); and 95 ± 2 mm Hg and 391 ± 12 bpm in rats whose femoral artery was ligated for 24 h (*n* = 8) (*P* > 0.05 vs. control). Typical traces and average data in Figure 5 show 24 h of femoral occlusion significantly increased the responses of MAP and HR evoked by muscle contraction. Moreover, Figure 5 shows MAP and HR responses to muscle contraction after blocking TRPA1 with arterial injection of 3 mg of HC-030031 in control rats and ligated rats. HC-030031 significantly inhibited MAP and HR responses induced by muscle contraction in control and ligated rats. In control rats, the MAP and HR responses were 16 ± 2 mmHg and 19 ± 3 bpm with saline injection; and 10 ± 2 mmHg and 10 ± 1 bpm with HC-030031 treatment (*P* < 0.05, HC-030031 treatment vs. saline control for both MAP and HR responses). In ligated rats, the MAP and HR responses were 27 ± 3 mmHg and 32 ± 3 bpm with vehicle injection; and 11 ± 2 mmHg and 11 ± 4 bpm with HC-030031 treatment (*P* < 0.05, HC-030031 treatment vs. vehicle control for both MAP and HR responses). The attenuating effects of HC-030031 were greater in ligated rats than in control rats (MAP and HR: 59 and 67% in ligated rats vs. 38 and 49% in control rats, *P* < 0.05. *n* = 8 in each group). No significant difference was observed in tension response in two groups. In six control rats, intravenous injection of HC-030031 failed to attenuate BP response to contraction (MAP response: 18 ± 3 mmHg before HC-030031 and 17 ± 2 mmHg after HC-030031, *P* > 0.05).

#### RSNA, BP, and HR responses to passive tendon stretch after blocking of TRPA1

**Figure 6:**
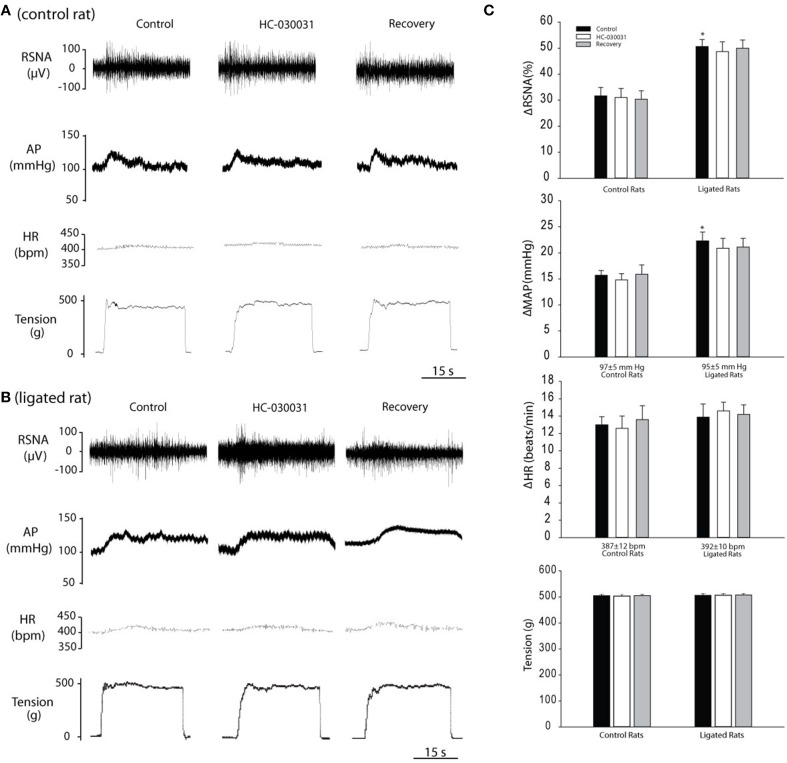
**Changes in RSNA, MAP and HR in response to passive tendon stretch after an inhibition of TRPA1 by 3 mg of HC-030031 (TRPA1 antagonist)**. Passive tendon stretch was evoked by loading ~500 g of muscle tension on the triceps surae muscle. **(A,B)** Typical traces obtained from a control rat and ligated rat. **(C)** Average data. After blocking TRPA1, responses of RSNA, MAP and HR to muscle stretch were observed in control rats (*n* = 10) and rats whose femoral artery was ligated for 24 h (*n* = 12). Note that blocking TRPA1 failed to attenuate the RSNA, MAP, and HR responses to muscle stretch in both control rats and ligated rats. Values are means ± SEM. ^*^P < 0.05 indicates ligated rats vs. control rats.

There were no significant differences in baseline values for MAP and HR in control rats (*n* = 10) and ligated rats (*n* = 12). Baseline MAPs and HRs were 97 ± 5 mm Hg; 387 ± 12 bpm in control rats; and 95 ± 5 mm Hg; 392 ± 10 bpm in ligated rats (*P* > 0.05 vs. control). Typical traces and average data in Figure 6 demonstrate RSNA, MAP, and HR responses to muscle stretch during each intervention. Before blocking TRPA1, significantly higher RSNA and MAP responses were observed in the ligated rats vs. the control rats. No significant HR responses were seen in the two groups during stretch. Blocking TRPA1 with arterial injection of 3 mg of HC-030031 did not elicit significant changes in RSNA, MAP and HR responses during muscle stretch in either the control rats or the ligated rats. i.e., in ligated rats, RSNA, and MAP responses induced by ~500 g of muscle tension were 49 ± 3% and 22 ± 3 mm Hg in control, and 48 ± 4% and 21 ± 3 mmHg (*P* > 0.05 vs. control) after 3 mg of HC-030031.

## Discussion

The purpose of the present study was to determine whether TRPA1 on primary muscle afferent nerves contributes to the enhanced sympathetic responsiveness elicited by femoral artery ligation. The results of this study have shown that that 24 and 72 h of femoral artery occlusion significantly amplifies the protein levels of TRPA1 in lumbar DRGs of ligated limbs, and that increased expression of TRPA1 selectively appears within DRG neurons that supply C-fiber afferents but not A-fiber afferents. In addition, stimulation of TRPA1 with injection of AITC into the arterial blood supply of the hindlimb muscles evokes greater increases in RSNA and BP in ligated rats than in control rats. This result further supports the idea that femoral artery occlusion upregulates the expression of TRPA1 in muscle afferents nerves. Moreover, our data demonstrate that blocking TRPA1 attenuates BP and HR responses during muscle contraction to a greater degree in ligated rats than those responses in control rats. In contrast, blocking TRPA1 fails to attenuate SNA and BP responses during passive tendon stretch in both groups. Accordingly, the findings of this study suggest that enhanced TRPA1 in muscle afferent nerves plays a role in regulating amplified sympathetic responsiveness after femoral artery occlusion via the metabolic component of the exercise pressor reflex.

Our prior work has suggested that femoral artery ligation increases expression of metabolite receptors (i.e., ASIC3 and P2X3) in DRG and there are insignificant differences in the levels of these receptors in rats with 24 h of ligation and in rats with 72 h of ligation (Liu et al., 2010, 2011). Likewise, the magnitudes of BP response to muscle contraction were observed to reach at a similar degree in rats with 24 h of ligation and in rats with 72 h of ligation (Tsuchimochi et al., 2010; Lu et al., 2012). Accordingly, in the current study we examined the effects of 24 h of ligation on the SNA, BP, and HR responses to AITC and to muscle contraction/stretch. However, it should be noted that TRPA1 expression in DRG of rats with 72 h of ligation appears to be slightly greater as compared with 24 h of ligation in the current study. It would be very likely to obtain optimal results if we have examined the reflex experiments 72 h post ligation. Another possibility is that an increased TRPA1 activity after 24 h of the femoral ligation is independent of its protein expression. Nonetheless, results of our current study have demonstrated that 24 h of ligation has greater effects on the reflex responses to arterial injection of AITC and HC-030031.

The current data show that 24 h femoral artery occlusion increased the protein levels of TRPA1 and amplified the SNA and BP responses to stimulation of AITC as compared with controls. One potential mechanism by which TRPA1 plays a regulatory role is a functional interaction of proteinase-activated receptor-2 (PAR2) and TRPA1 in DRG neurons. Co-localization of TRPA1 with PAR2 has been found in rat DRG neurons and the activation of PAR2 has been reported to increase TRPA1 induced currents in DRG neurons (Dai et al., 2007). In addition, a prior study has shown that the mRNA expression of PAR2 increased by 1.9-fold in adductor muscles of mice whose femoral artery was ligated for a day (Milia et al., 2002).

In a prior study using the patch clamp methods, AITC from 10 to 1000 μM were successively applied to the same DRG neuron and the data of this study demonstrated that EC50 value for AITC is 173 μM and the saturating concentration is about 1 mM (Raisinghani et al., 2011). Brief application (20–50 s) of AITC at 200 μM has been shown to induce responses that were readily reversible, but continuous application of higher concentrations (>500 μM) of AITC induced a complete desensitization of the current response (Raisinghani et al., 2011). A recent study has also shown that in healthy decerebrated rats, intra-arterially injection of AITC (10–50 μg/kg body weight) into the hindlimb muscle circulation led to increases in SNA via a reflex pathway (Koba et al., 2011). Also, a dose-response relation was observed after those dosages of AITC were injected into the femoral artery (Koba et al., 2011). Considering that AITC was dissolved in the 0.2 ml of saline and its molecular weight is 99.15, the concentrations of AITC used in this *in vivo* study were ~0.5–2.5 mM (Koba et al., 2011). Because AITC injected into the muscle tissue via femoral artery can be partly metabolized we assumed that 10–50 μg/kg of AITC were effective to stimulate TRPA1 with minimal desensitization. Furthermore, this prior study has demonstrated that TRPA1 plays a role in regulating SNA via stimulating the metabolic component of the exercise pressor reflex. Therefore, in the current study, the similar dosages AITC was selected to stimulate TRPA1 on muscle afferent nerves and our results show that greater SNA and BP response are evoked in ligated rats after arterial injection of the same dosage of AITC into the hindlimb muscles than those responses in control rats. However, it is noted that femoral ligation did not significantly amplified HR response to AITC. This is consistent with our previous results showing that HR response to activation of other metabolic receptors (i.e., ASIC3 and P2X3) is not significantly altered by femoral ligation (Liu et al., 2010, 2011).

In addition, prior studies have demonstrated that TRPA1 knockout mice displayed behavioral deficits in sensing noxious cutaneous mechanical pain (Kwan et al., 2006, 2009). TRPA1 is considered to likely play a role in mediating mechanical hypersensitivity (Brierley et al., 2011). Given that thin fiber muscle afferent nerves (group III & IV) innervating skeletal muscles are activated by contraction-induced mechanical and metabolic stimuli to elicit reflex increases in SNA and BP during the exercise pressor reflex (Mitchell et al., 1983; Kaufman and Forster, 1996), we also examined the effects of blocking TRPA1 on the SNA, BP and HR responses evoked by static muscle contraction and passive tendon stretch. Our data show that femoral ligation significantly amplified BP and HR responses to muscle contraction (stimulation of both mechanically and metabolically sensitive muscle afferent nerves). Furthermore, blocking TRPA1 attenuated BP and HR responses to muscle contraction to a greater degree in ligated rats than those responses in control rats. It is speculated that a larger level of TRPA1 was likely attenuated after injection of HC-030031 into the hindlimb. This is consistent with results demonstrating that expression of TRPA1 in DRG neurons appeared to be greater in ligated rats than that in control rats. On the other hand, blocking TRPA1 failed to attenuate SNA, BP, and HR responses during passive tendon stretch (stimulation of mechanically sensitive muscle afferent nerves) in both groups. Thus, it would appear that stimulation of TRPA1 does not activate the mechanical component of the exercise pressor reflex.

What are endogenous muscle metabolites within contracting muscles to stimulate TRPA1 still needs to be determined. In addition to pungent chemicals found in nature, endogenously generated molecules such as bradykinin, reactive oxygen species, and 4-hydroxynonenal that are produced during inflammation and oxidative stress, respectively, can activate TRPA1 (Bandell et al., 2004; Trevisani et al., 2007; Bessac et al., 2008). Acid phosphate, bradykinin and arachidonic acid, which are accumulated in contracting muscles, are considered as potential endogenous stimuli engaged in the exercise pressor reflex (Koba et al., 2011). Furthermore, recent studies from our laboratory and others support the concept that muscle metabolites can sensitize mechanically sensitive afferents and increase mechanoreflex-mediated SNA response (Li and Sinoway, 2002; Koba et al., 2007; Cui et al., 2008; Gao et al., 2008). Sensitization of the mechanoreflex would not occur during passive muscle stretch as metabolites are not accumulated during this maneuver. This would account for why blockade of TRPA1 was ineffective at attenuating the response to stretch but might still play a role in the sensitization of the mechanoreflex during contraction (when metabolites accumulate). Bradykinin has been reported to modulate the exercise pressor reflex via kinin B2 receptor (Pan et al., 1993). Femoral artery occlusion amplifies SNA and BP responses to stimulation of stimulation of mechanically sensitive muscle afferent nerves and increased responses are attenuated by blocking kinin B2 receptor (Leal et al., 2013; Lu et al., 2013). Thus, we speculate that some muscle metabolites and their respective receptors such as bradykinin/kinin B2 receptor may play a role in regulating the exercise pressor reflex via TRPA1 engagement as stimulus as well as modulator after femoral artery occlusion.

TRPA1 has a similar structure to TRPV1 receptor, but with numerous ankyrin repeats in its amino (N) terminal (Jaquemar et al., 1999). TRPA1 has been shown to be co-expressed with TRPV1 (Story et al., 2003). Many agonists have been identified to activate both TRPA1 and TRPV1 receptors (Koizumi et al., 2009; Okumura et al., 2010). AITC can activate porcine TRPV1 (Ohta et al., 2007). Note that application of AITC in the micromolar range did not significantly affect currents recorded in TRPV1-transfected HEK293 cells. However, TRPV1 contributes to the DRG neuron responses to 3 mM of mustard oil (Everaerts et al., 2011). Likewise, a previous study has shown that cell membrane staining of TRPA1 increased upon 1 min treatment of 1 μM of capsaicin (Schmidt et al., 2009). Also, this prior study has demonstrated that activation of TRPV1 by capsaicin that was accompanied with localized calcium influx acutely increased TRPA1 membrane surface expression while TRPV1 levels were unchanged (Schmidt et al., 2009). Our published work showed that a higher density of TRPV1 immunostaining was induced within DRG neurons of rats with 24 h of femoral artery occlusion when compared with that in control rats, and that the arterial occlusion insult induced greater peak of inward current amplitudes in DRG neurons to capsaicin (Xing et al., 2008). Thus, we speculate that artery occlusion-enhanced expression and responses of TRPV1 in DRG neurons likely induced a greater calcium influx and increased TRPA1 membrane surface expression in engagement in the role played by TRPA1 observed in our current study.

In summary, the data of the present study have shown that the femoral artery occlusion significantly increases the levels of TRPA1 protein in lumbar DRG neurons. The enhanced expression of TRPA1 is specifically observed in DRG neurons supplying C-fiber muscle afferent nerves. Stimulation of TRPA1 in DRG neurons with AITC evokes greater increases in SNA and BP in rats with femoral occlusion. In addition, blocking TRPA1 in muscle afferent nerves significantly attenuates SNA and BP responses evoked by stimulation of metabolically sensitive muscle afferent nerves, but not by stimulation of mechanically sensitive muscle afferent nerves. Overall, our findings suggest that TRPA1 in muscle afferent nerves plays an important role in augmented sympathetic responsiveness via the metabolic component of the exercise pressor reflex when blood supply to the hindlimb muscles is insufficient as observed in PAD.

## Grant support

This study was supported by NIH P01 HL096570 & NIH R01 HL090720 and American Heart Association Established Investigator Award 0840130N.

### Conflict of interest statement

The authors declare that the research was conducted in the absence of any commercial or financial relationships that could be construed as a potential conflict of interest.
